# RD-Metabolizer: an integrated and reaction types extensive approach to predict metabolic sites and metabolites of drug-like molecules

**DOI:** 10.1186/s13065-017-0290-4

**Published:** 2017-07-18

**Authors:** Jiajia Meng, Shiliang Li, Xiaofeng Liu, Mingyue Zheng, Honglin Li

**Affiliations:** 1 State Key Laboratory of Bioreactor Engineering, Shanghai Key Laboratory of New Drug Design, School of Pharmacy, East China University of Science and Technology, Shanghai, 200237 China; 2 Shanghai Key Laboratory of Chemical Biology, School of Pharmacy, East China University of Science and Technology, 130 Meilong Road, Shanghai, 200237 China; 3 Drug Discovery and Design Center, Shanghai Institute of Materia Medica, Chinese Academy of Sciences, Shanghai, 201203 China

**Keywords:** Sites of metabolism (SOMs), Metabolites, Reaction SMARTS patterns, 2D fingerprint similarity

## Abstract

**Background:**

Experimental approaches for determining the metabolic properties of the drug candidates are usually expensive, time-consuming and labor intensive. There is a great deal of interest in developing computational methods to accurately and efficiently predict the metabolic decomposition of drug-like molecules, which can provide decisive support and guidance for experimentalists.

**Results:**

Here, we developed an integrated, low false positive and reaction types extensive metabolism prediction approach called RD-Metabolizer (Reaction Database-based Metabolizer). RD-Metabolizer firstly employed the detailed reaction SMARTS patterns to encode different metabolism reaction types with the aim of covering larger chemical reaction space. 2D fingerprint similarity calculation model was built to calculate the metabolic probability of each site in a molecule. RDKit was utilized to act on pre-written reaction SMARTS patterns to correct the metabolic ranking of each site in a molecule generated by the 2D fingerprint similarity calculation model as well as generate corresponding structures of metabolites, thus helping to reduce the false positive metabolites. Two test sets were adopted to evaluate the performance of RD-Metabolizer in predicting SOMs and structures of metabolites. The results indicated that RD-Metabolizer was better than or at least as good as several widely used SOMs prediction methods. Besides, the number of false positive metabolites was obviously reduced compared with MetaPrint2D-React.

**Conclusions:**

The accuracy and efficiency of RD-Metabolizer was further illustrated by a metabolism prediction case of AZD9291, which is a mutant-selective EGFR inhibitor. RD-Metabolizer will serve as a useful toolkit for the early metabolic properties assessment of drug-like molecules at the preclinical stage of drug discovery.

**Graphical abstract:**
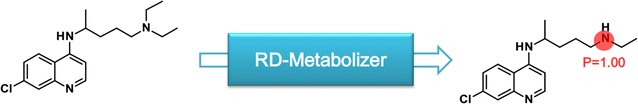
A visual example of the metabolic site and the corresponding metabolite of Chloroquine predicted by RD-Metabolizer

## Introduction

It is significant to know how drug candidates are metabolized in the body at early stages of the drug discovery process, because both the drug safety and efficacy profiles are greatly affected by human metabolism [1]. The drug-like molecules can be either metabolized into their active forms to actually interact with the therapeutic targets, or converted into inactively execrable metabolites [1]. In addition, the metabolic modifications can also bring toxicity, which is one of the major reasons for failure in drug development. Furthermore, metabolic liability is also related to other critical issues, for example drug–drug interactions, food–drug interactions and drug resistance [2–4]. Therefore, it is of great importance to determine the metabolic properties of the drug candidates earlier. However, experimental approaches for determining those properties are usually expensive, time-consuming and labor intensive [5]. Thus, there is a great deal of interest in developing computational methods to accurately and efficiently predict the metabolic decomposition of drug-like molecules [6–9].

The investigations of SOMs and structures of metabolites are two main research directions of computer-aided metabolism prediction methods, which can provide decisive support and guidance for experimentalists [10]. The prediction methods of SOMs usually have higher prediction accuracy. For example, MetaSite [11], a commercial software package, utilizes GRID-derived molecular interaction fields (MIFs) of protein and ligand, protein structural information, and molecular orbital calculations to estimate the likelihood of metabolic reaction at a certain atom position, with a success rate of 85% for tagging a known SOM among the top-2 ranked atom positions. Rydberg et al. [12–14] implemented SMARTCyp as a fast SOMs predictor. The predictor contains a reactivity lookup table of pre-calculated density functional theory (DFT) activation energies for plenty of ligand fragments that are undergoing a CYP3A4 or CYP2D6 mediated transformation. SMARTCyp performs a fast reactivity lookup for the query compound, in conjunction with a topological accessibility descriptor to provide a final SOM ranking. As a result, SMARTCyp identified 76% of SOMs over a dataset of 394 compounds with the top-2 metric. RegioSelectivity (RS)-predictor is developed by Zaretzki et al. [15, 16], which employs a set of 392 quantum chemical atom-specific and 148 topological descriptors, and a support vector machine (SVM)-like ranking in combination with a multiple instance learning method to determine potential SOMs. Using the top-2 metric, 78% of SOMs were identified over a test set of 394 compounds. MetaPrint2D [17–20] identifies the reaction center atoms for the substrates recorded in biotransformation database through the maximum common substructure method. Each substrates atom and reaction center atom is encoded in a six-level topological fingerprint. Therefore, two fingerprint databases are yielded in this process. For a query molecule, it is firstly converted into fingerprints, then the fingerprint of each atom is matched against the above two fingerprint databases. By comparing the similarity of fingerprint, the number of hits in each database can be counted. Finally, the metabolic likelihood of each atom in the query molecule is derived. About 70–80% of SOMs in the test compounds are correctly predicted among the three highest-scored atom positions. Quite impressive results can be obtained by these computational methods, however, most of these approaches are limited to CYP450 catalyzed reactions, and only labile sites rather than structures of metabolites can be predicted. Moreover, predicted SOMs are not equivalent to identifying the correct biotransformation that would take place at a certain atom position, and they provide no information about which reaction type will take place. Therefore, these limitations make it difficult to draw any quantitative conclusions on the metabolic liability of a certain molecule [10]. Besides, these methods are also less suitable for routine use to support experimental identification of metabolites.

Predicting the structures of metabolites by computational approaches in advance can decisively help medicinal chemists analyze the experimentally-determined mass spectrometry (MS) data or liquid chromatography/tandem mass spectrometry (LC–MS/MS) data to pinpoint the actual SOMs [21]. However, only very few computational methods to predict structures of metabolites have been developed so far. These prediction approaches are usually clustered into three categories: expert systems, fingerprint-based data mining approaches and combined approaches. Expert systems mainly employ generic metabolic rules derived by expert to predict structures of metabolites. Typical examples of expert systems are META [22–24], MetabolExpert [25], Meteor [26], SyGMa [27], TIMES [28]. For the fingerprint-based data mining approaches, MetaPrint2D-React [18], an extension of MetaPrint2D, is a typical and representative method. It is and allows users to predict structures of metabolites on the basis of generic metabolic reaction rules. Tarcsay et al. [29] firstly adopt the best setup of the expert system MetabolExpert [25] to generate possible metabolites for the query compound. Then the docking program GLIED [30] as a postprocessing filter is employed to reduce the false positive rate. This combined approach brings a success rate of 69% for identifying the correct metabolites among the three highest-ranked structures. Although these methods have an advantage in speed or correctly generating structures of metabolites, there still exist several challenges. The main drawback of expert system is the combinatorial explosion problem, because all possible combinations of metabolic rules permitted by the reaction rule sets are considered. The disadvantage of fingerprint-based data mining method is that generic metabolic transformation rules are so simple that they cannot describe complex reaction types and cannot cover larger chemical reaction space. The method combined with docking is impractical for many applications, due to its time-consuming and structure-dependent features.

The main contribution of this work is a description of Reaction Database-based Metabolizer (RD-Metabolizer), an integrated, low false positive and reaction types extensive approach for predicting metabolic sites and metabolites of drug-like molecules. In order to cover larger chemical reaction space, the detailed reaction SMARTS patterns were firstly employed to describe simple and complex reactions recorded in the biotransformation databases. 2D fingerprint similarity calculation model was built to calculate the metabolic probability of each site in a molecule. Meanwhile, RDKit [31], an open-source chemical information software, was utilized to act on pre-written reaction SMARTS patterns to correct the metabolic ranking of each site in a molecule and generate corresponding structures of metabolites. In comparison studies, RD-Metabolizer performed slightly better than or at least as good as several widely used SOMs prediction methods in terms of SOMs prediction accuracy. And compared with other metabolite prediction method, the number of false positive metabolites generated by RD-Metabolizer was also obviously reduced. A specific metabolism prediction example of AZD9291 [32] further indicated its robustness in SOMs identification and metabolites generation, and also confirmed its potential applications for metabolism prediction.

## Experimental methods

**Fig. 1:**
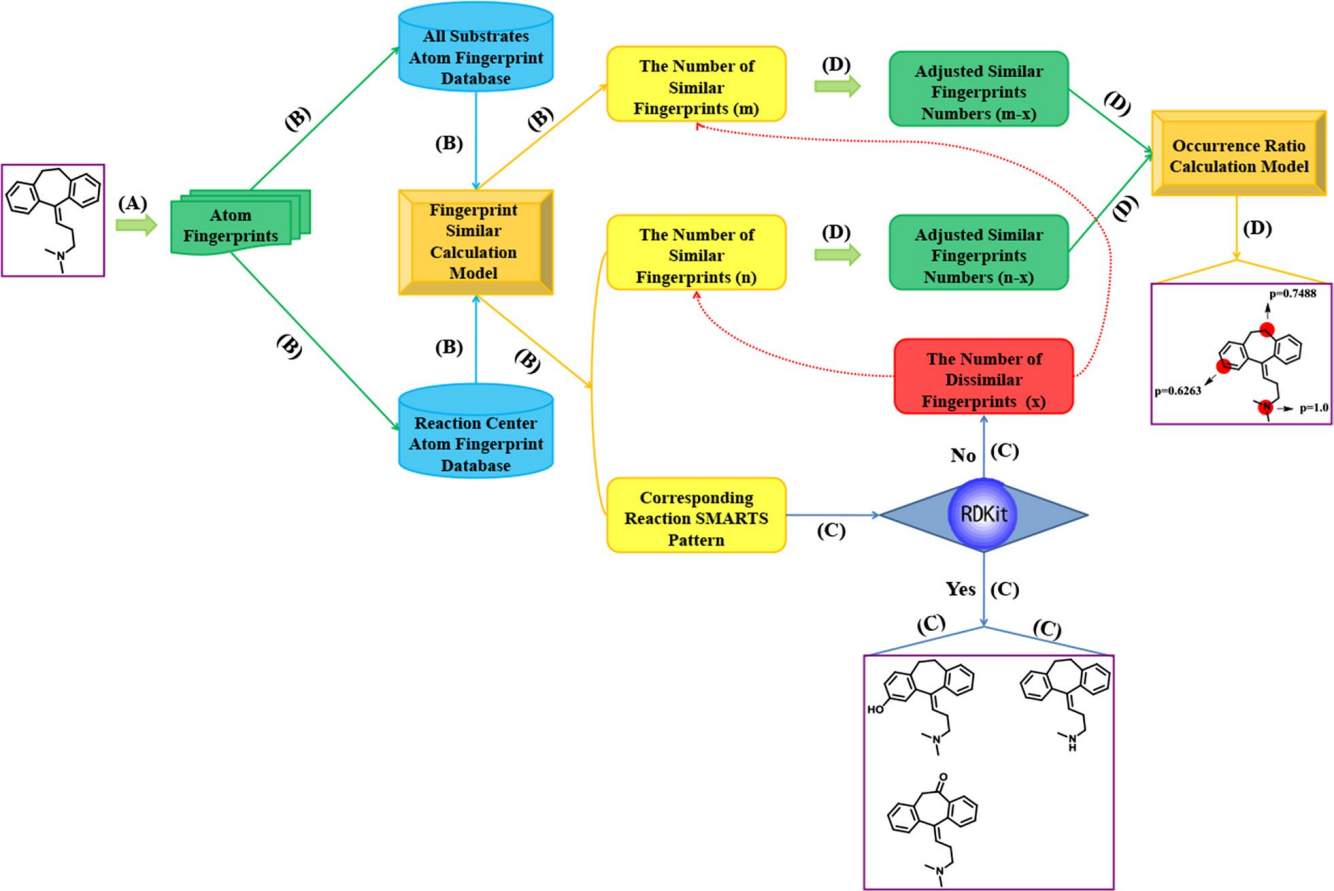
Schematic representation of RD-Metabolizer workflow for SOMs and metabolites prediction. *(A)* Convert query compound to topological atom fingerprints; *(B)* search the two fingerprint databases by 2D fingerprint similarity calculation model, thus respectively get the number of similar fingerprints from two databases and the corresponding reaction SMARTS patterns from the reaction center topological atom fingerprint database; *(C)* check if RDKit can act on the reaction SMARTS patterns, then count the number of dissimilar fingerprint and generate corresponding metabolite structures of each site; *(D)* adjust the number of similar fingerprints and calculate occurrence ratio of each site

The framework of RD-Metabolizer is illustrated in Fig. 1. Firstly, the query molecule is converted into suitable fingerprint to fit for the fingerprint-based similarity calculation model. Secondly, the fingerprint of each atom in the query molecule is matched against two topological atom fingerprint database. One database comprises all the atomic fingerprints of the substrates, and the other one contains all the reaction centers that marked with reaction SMARTS patterns. By calculation of the fingerprint similarities, the total numbers of similar fingerprints in the above two fingerprint databases are counted respectively, meanwhile, the corresponding reaction SMARTS patterns are obtained from the latter database. Thirdly, because the calculated similar fingerprints do not always represent the similar chemical environment of the corresponding sites, RDKit is firstly applied to check whether the calculated similar fingerprints are indeed similar with each other. If the structures of metabolites can be generated by RDKit through manipulating the reaction SMARTS patterns obtained from the previous step, the calculated similar fingerprints are proved to be true similar pairs. If not, they are identified as dissimilar fingerprints, and the number is counted. Finally, the reaction occurrence ratio of each site in the query molecule is calculated and normalized. Further details of the RD-Metabolizer workflows are described below.

### Data sources

Dataset used in the present study was extracted from MDL metabolic reaction database [33] and integrity database [34], which both included metabolic transformations of xenobiotic compounds harvested from the literatures. The dataset generation procedure was as follows: (1) repeated reactions were handled (only used single-step and unique reactions to avoid data redundancy); (2) molecules in reactions must have a complete chemical structure, thus reactions that reactant or product had “R” substituents or free radical were excluded; (3) reactions that reactant or product was invalid were processed (i.e. reactant or product was labeled with “No Structure”); (4) chelation reactions and reactions with ambiguous reaction centers were also excluded (No reaction SMARTS pattern could express these reactions); (5) reactions that reactant or product was a single element (i.e. metallic element) were removed. Finally, 63,620 individual metabolic reactions were retained as the metabolic reaction dataset for further study.

### Preparation of test sets

We randomly selected 425 different substrate molecules from the metabolic reaction dataset to be internal test set (test set 1). After remove the metabolic reaction records of these 425 substrate molecules, the rest of the metabolic reaction records were used to generate the two topological fingerprint databases required by RD-Metabolizer. The external test set (test set 2) compiled by Zaretzkiet et al. [16] was used for further method validation. For the external test set, some structures were found identical to those in our training sets, and thus removed. As a result, the external test set contained 173 compounds. Besides, all the test compounds were carefully checked to ensure the correctness of their 2D structures. Wrong structures were corrected by manually searching different databases, such as DrugBank [35] and PubChem [36].

### Identification of SOMs and generation of reaction SMARTS patterns

For the databases, all data are curated in the form of metabolic reactions and no SOMs are explicitly reported, so the SOMs information needs to be derived. A SOM refers to the place in a molecule where the metabolic reaction occurs. In order to identify a SOM, the exact or determinable biotransformation mechanism needs to be known. However, many biotransformation mechanisms of metabolic reactions are still beyond understanding and information on SOMs is very sparse, especially for enzymes other than CYP450s [37]. There are two main methods to identify SOMs. One is maximum common substructure method. This method firstly examines the maximum common substructure of the substrate and the product, and then deviations from the maximum common substructure in either substrate or product are identified as reaction sites [18]. The other method is based on the calculation of activation energies of ligand fragments. It is reported that the lower the activation energies are, the more likely a site is to be metabolized [12]. In our study, for simple biotransformation reactions, we manually compared structures of reactant and product in each pair of metabolic reactions to determine SOMs. Any positions of a reactant molecule where a heavy atom was added, removed, or altered were intuitionistic regarded as SOMs. For example, for O,N,S-demethylation reactions, we took heteroatom (O,N,S) as metabolic reaction center atom. However, for some complex biotransformation reactions, we could not directly determine their SOMs by visual comparison. Therefore, we extracted the SOMs according to the structural changes of reactant and product represented in reaction SMARTS patterns. Reaction SMARTS pattern is analogous to Daylight SMARTS language [38] enabling description of biotransformation reactions. Reaction SMARTS pattern can describe partial structures of reactant and product molecules, and specify atom mappings of structures. Some examples of simple and complex biotransformation reactions by means of reaction SMARTS patterns to identify SOMs are shown in Table 1.

**Table 1:**
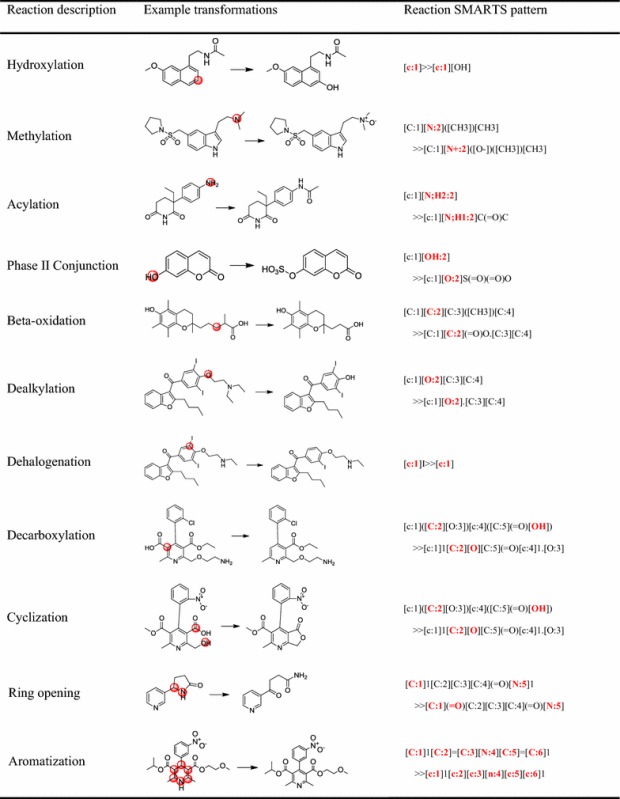

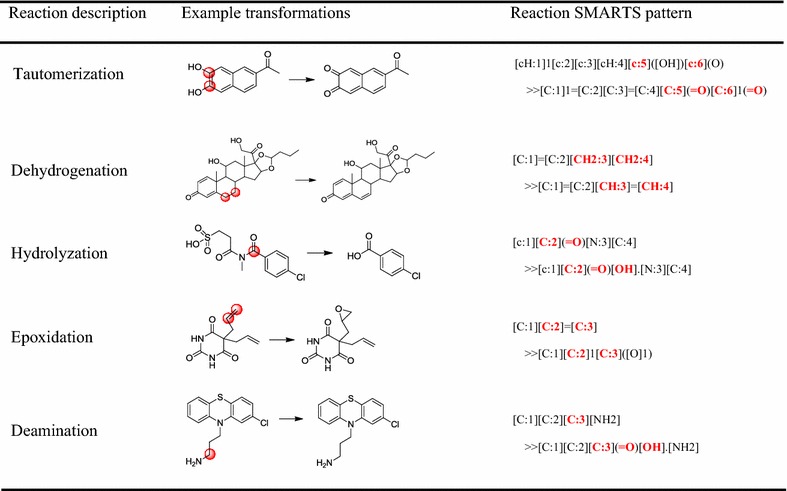
Examples of identifying SOMs of simple and complex biotransformation reactions through reaction SMARTS pattern

Bold red in the square brackets: atoms that have structural variations are represented in the reaction SMARTS pattern. Red circle in molecule: based on the reaction SMARTS patterns, the corresponding reaction centers are labeled

### Generation of fingerprint databases

**Fig. 2:**
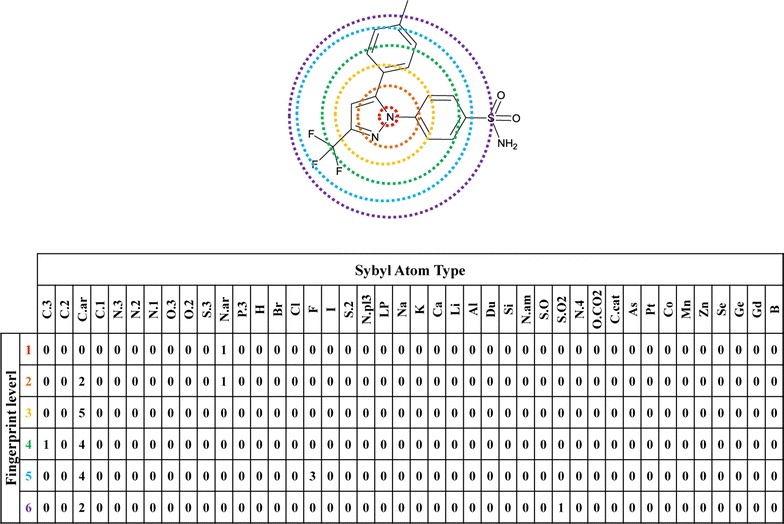
Construction of six layers topological atom fingerprint. The starting layer is an N atom (sybyl atom type: N.ar) in the *red circle* . The successive layers range from *orange* to *yellow*, *green*, *blue*, and *violet*. Atoms lying far away from the six-layer are not considered. Below the fingerprint matrix represents the counts of SYBYL atoms types and another 9 atoms that involved in metabolic reactions at each layer. The *rows* are colored according to the same color scheme of the figure above

For the purpose of modeling, we need two fingerprint databases: topological atom fingerprint database of all substrates and topological atom fingerprint database of all reaction centers with reaction SMARTS patterns. Molprint2D fingerprint [39, 40] was used in the present study because of its ability in representing the chemical environment occupied by atoms and satisfying requirement of quantitative calculation. The generation process of two fingerprint databases was presented below. Firstly, Molprint2D fingerprints of all substrates were generated by Pipeline Pilot 7.5 (Accelrys San Diego, California) with the fingerprint layer of each atom set to six. For the molecules whose fingerprint layers of some atoms were less than six, the character “A” was added manually to the missing layers of the atoms in those molecules to meet the requirement of quantitative calculation. Secondly, the topological atom fingerprint database of all substrates was generated by a python script, which counts occurrence frequencies of atom types in each layer. In this work, atom types were made up of the 33 Tripos mol2 atom types [41] and other atom types that presented in the metabolic reactions, such as As, Pt, Co, Mn, Zn, Se, Ge, Sn, Gd and B. Celecoxib [42], a non-steroidal anti-inflammatory drug, was selected as an example of the construction of six layers topological atom fingerprints (Fig. 2). Thirdly, SOMs of all substrates were identified by using the method described above, then Molprint2D fingerprints of SOMs were extracted and correspondingly compiled reaction SMARTS patterns were subsequently added to the next layer. Molprint2D fingerprints of SOMs and corresponding reaction SMARTS patterns were both stored in text files. Moreover, the topological atom fingerprint database of all reaction centers with reaction SMARTS patterns was also built by a python script.

### Occurrence ratio calculator

After generation of the topological atom fingerprints for the query compound, the fingerprint of each atom in query compound was matched against the two fingerprint databases. In the present work, we built a 2D fingerprint similarity calculation model to calculate the metabolic occurrence ratio of each atom in the query compound. The similarity calculation model was composed of three similarity operators, namely *Exact match operator*, *Soergel metric operator* [43, 44] and *Hamming metric operator* [45], to compare the fingerprint matrices. In order to compute fast and ensure the existence of cored substructures that are key for determining whether the two fingerprint are similar, the *Exact match operator* was firstly performed, which requires the layers in two fingerprint matrices to be exactly the same (top three layers were adopted in our method), thus the fingerprints that do not match top three layers can be rejected quickly. Then, the *Soergel metric operator* and the *Hamming metric operator* were employed. Finally, the number of similar fingerprints in each database was counted.

The Soergel metric and the Hamming metric between two fingerprints *a* and *b*, for the *j*_th_ row, were defined as Eqs. (1) and (2). The finally scoring function can be represented by the sum of weighted scores for the each level, which defined as Eq. (3).

1 $$ d_{j} = 1.0 - \frac{{\sum\nolimits_{n = 1}^{33} {F_{j,n}^{a} F_{j,n}^{b} } }}{{\sum {\left[ {\left( {F_{j,n}^{a} } \right)^{2} + \left( {F_{j,n}^{b} } \right)^{2} - F_{j,n}^{a} F_{j,n}^{b} } \right]} }} $$

2 $$ d_{Ham,j} = \sum\limits_{n = 1}^{33} {\left| {F_{j,n}^{a} - F_{j,n}^{b} } \right|} $$

3 $$ d_{total} = \sum\limits_{j = 0}^{5} {{\Delta} \lambda_{j} \left( {d_{j} \times d_{Ham,j} } \right)} $$

where $\Delta \lambda_{j}$ is a weighting coefficient that can be used to adjust the significance of each row of the fingerprints and formulated as following:

4 $${ \Delta} \lambda = \frac{2}{{\lambda_{total} }}\left[ {\left( {\frac{\lambda }{{e^{\lambda - 1} }}} \right) + \left( {\frac{{\lambda_{total} - 1}}{2\lambda }} \right)} \right] $$

where *λ* ≥ 1 and the total number of levels, *λ*_*total*_ = 6 [43].

In this study, two fingerprints were considered to be similar if the scoring function *d*_*total*_ ≤ 3.5 [*d*_*total*_ was range from 0 (identity) to ∞ (maximum diversity)]. When *d*_*total*_ ≤ 3.5, the false negatives were the least for a set of tested fingerprints.

The calculations of occurrence ratios and normalized occurrence ratio are the same as those applied by Boyer et al. [18] and defined as Eqs. (5) and (6).

5 $$ r_{i} = (n - x)/(m - x) $$

6 $$ p = r_{i} /\hbox{max} (r_{i} ) $$

where *m* is the number of similar fingerprints that was searched from the topological atom fingerprint database of all substrates for the *i*th atom; *n* is the number of similar fingerprints that was searched from the topological atom fingerprint database of all reaction centers for the *i*th atom, and *x* represents the number of dissimilar fingerprints, which is the corrected result by calling RDKit to manipulate the pre-written reaction SMARTS patterns.

In our study, we used the following division rules to distinguish the metabolic possibilities [18]: very unlikely, 0 ≤ *p* < 0.15; unlikely, 0.15 ≤ *p* < 0.33; likely, $0.33 \le p < 0.66$; very likely, 0.66 ≤ *p* < 1.00.

## Results and discussion

In order to correctly predict structures of possible metabolites of the query compound, SOMs should be correctly identified at first. Benefited by the combination of the 2D similarity calculation model and the pre-written reaction SMARTS patterns, the SOMs and metabolites prediction performance of RD-Metabolizer are investigated.

### Prediction of metabolic sites

There are two main methods to evaluate the prediction performance of SOMs: qualitative analysis and quantitative analysis [12, 16, 17, 46, 47]. Qualitative analysis mainly rely on visual inspection, namely, the predicted results of a method is compared with the known metabolic sites of the molecules. Quantitative analysis refers to the percentage of molecules for which at least one of the top *k* (usually *k* = 1–3) ranked sites is an experimentally observed SOM. However, this index often depends on the size of the molecules, and the number of metabolic sites, which will result in a tendentious prediction. Prediction of SOMs can be treated as a classification problem: each site in a molecule is either a metabolic site or not. Therefore, in order to overcome the bias of top *k* metric, an overall measurement index called area under the curve (AUC) of the receiver operating characteristic (ROC) for SOMs prediction assessment is proposed [17]. This method was also applied in our study.

**Fig. 3:**
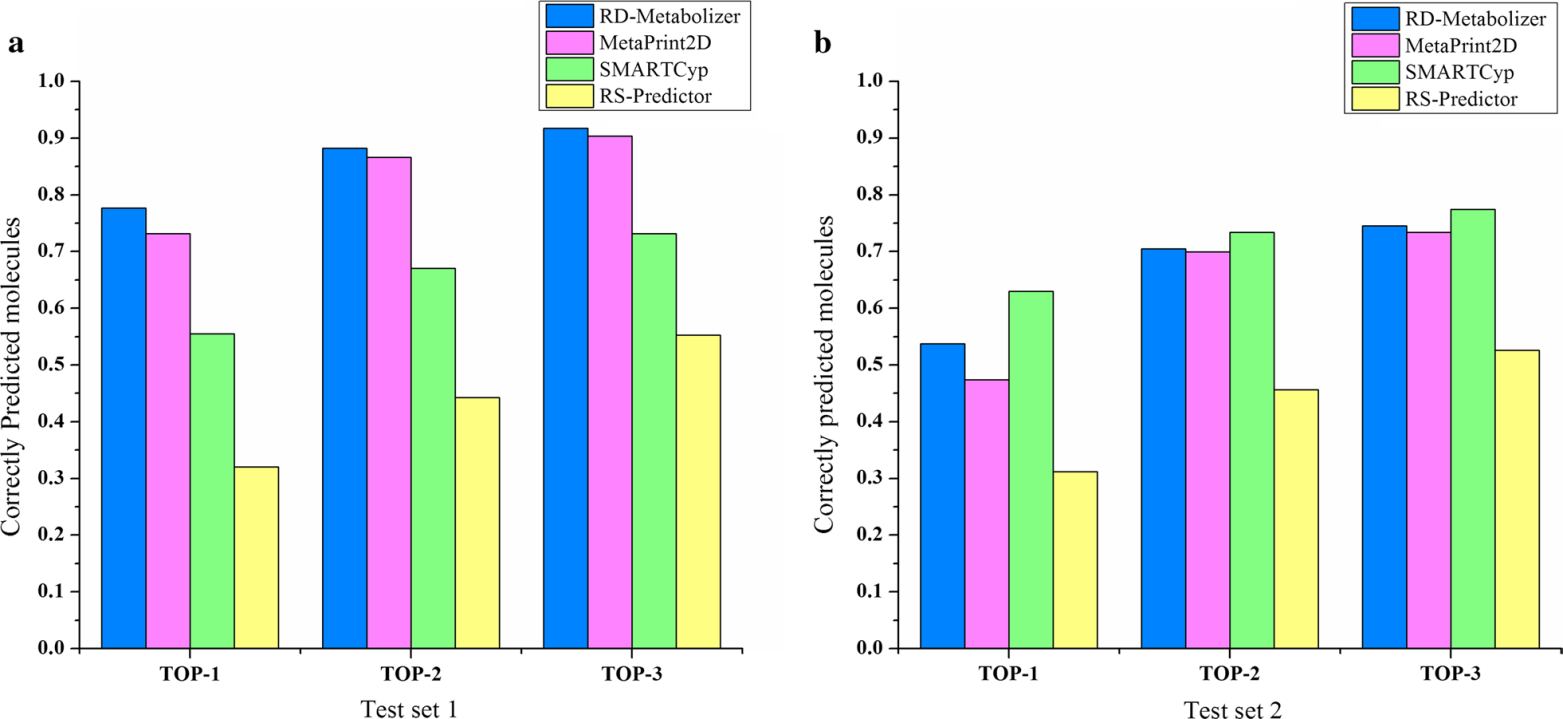
Comparison of SOM prediction performance of RD-Metabolizer and other predictors. Histograms show the correct prediction ratios **a** for test set 1 and **b** for test set 2 at the top-*k* (*k* = 1, 2, 3) metric, respectively

We compared the performance of RD-Metabolizer with some widely used SOMs prediction methods, such as MetaPrint2D (version 1.0), SMARTCyp (version 2.4.2) and RS-predictor (combined model). Default settings were used for the three methods. For test set 1, our method performed as well as MetaPrint2D, but better than SMARTCyp and RS-predictor at all the top three layers (Fig. 3a). Both RD-Metabolizer and MetaPrint2D are fingerprint-based data mining approaches that depend on the size of metabolite database, thus they have similar performances. The poorer prediction ability of SMARTCyp is mainly attributed to its limited range of reactions (only phase I redox reactions). Therefore, SMARTCyp is less sensitive to the polar groups, which are easily conjugated with the endogenous cofactors and occur phase II metabolism. Although RS-predictor established different CYP450 isoforms prediction models, we cannot know in advance which isoforms of CYP450 will participate in the metabolic reactions. Actually, one or more CYP450 isoforms may be involved in the metabolism of xenobiotic and endogenous compounds. That’s why we chose its combined model to compare with our method rather than a specific CYP450 isoform prediction model.

As for test set 2, the top-*k* (*k* = 1–3) prediction rates of RD-Metabolizer are better than MetaPrint2D and RS-predictor (Fig. 3b). Although the top-1 prediction rate of RD-Metabolizer for test set 2 is inferior to SMARTCyp, both top-2 and top-3 prediction rates of RD-Metabolizer are comparable with SMARTCyp. Compared to the top-2 and top-3 prediction rates, there may be three reasons causing the difference in the top-1 prediction rate of RD-Metabolizer and SMARTCyp. Firstly, the definition of SOMs between RD-Metabolizer (reaction SMARTS pattern-based) and SMARTCyp (mechanism-based) is different. For example, in the case of N-/O-dealkylation, RD-Metabolizer ranks the heteroatom higher than the carbon atom, while SMARTCyp takes the carbon atom that connect to the heteroatom as reaction center. Secondly, RD-Metabolizer is a fingerprint similarity-based method, and predictions cannot be performed about novel atomic sites where the topological fingerprint does not exist in the two databases we built. Thirdly, after examination, it is found that compounds in the test set 2 are mainly involved in phase I metabolism, while two fingerprint databases of RD-Metabolizer we built contain fingerprints of both phase I and phase II metabolic sites. Therefore, some polar sites of the compounds in test set 2 may bring impact on the final metabolic site rankings.

**Fig. 4:**
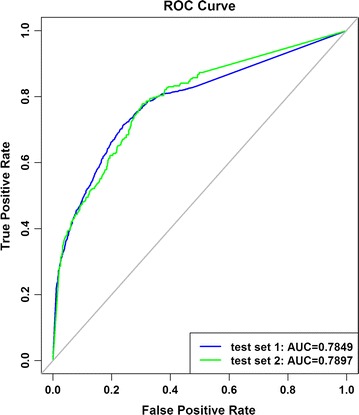
ROC curve of SOMs predictions for test set 1 (*blue*) and test set 2 (*green*). Test set 1 is the internal test set that contains 425 different randomly selected substrate molecules from the metabolic reaction dataset. Test set 2 is the external test set that contains 173 compounds and was previously compiled by Zaretzkiet et al. [16]

ROC curve was made for test set 1 and 2 to discuss the performance of RD-Metabolizer in terms of distinguishing metabolic sites from non-metabolic ones, and the corresponding overall AUC values were obtained (Fig. 4). Besides, the mean AUC and median AUC values were also calculated. The ROC curves obtained by our method are higher than the average diagonal, and the overall AUC values for test set 1 and 2 are close to each other (0.785 vs 0.790). Specifically, the mean AUC values for test set 1 and 2 are 0.811 and 0.831 respectively; meanwhile, the corresponding median AUC values are 0.852 and 0.913. Collectively, the AUC values demonstrated that our method has good performance in distinguishing metabolic sites from non-metabolic ones.

### Prediction of structures of metabolites

To quantitatively assess the performance of RD-Metabolizer in predicting structures of metabolites, we not only measured its ability to reproduce the experimentally determined metabolites (i.e. the recall) from the top-*k* (*k* = 1, 3) ranking list, but also measured the enrichment rates of these correct metabolites in the top-*k* (*k* = 1, 3) positions, namely the precision. Besides, *F*1-Measure was applied and served as comprehensive performance evaluation index. The corresponding calculation formulas are defined as following:

7 $$ top{\text{-}}k\text{:}recall = \frac{{The{\text{ number of }}real \, metabolites \, in \, the \, top{\text{-}}k \, position}}{{The{\text{ total number of experimental }}metabolites}} $$

8 $$ top{\text{-}}k\text{:}precision = \frac{{The{\text{ number of }}real \, metabolies \, in \, the \, top{\text{-}}k{\text{ position}}}}{{The{\text{ total number of }}predictive \, metabolites \, in \, the \, top{\text{-}}k{\text{ position}}}} $$

9 $$ top{\text{-}}k\text{:}F1{\text{-}}Measure = \frac{2 * precision\; * \;recall}{precision + recall} $$

At the same time, because the development of RD-Metabolizer was aimed at decreasing the number of false positive metabolites in predictions, we counted the total numbers of false positive metabolites for all molecules in the test set, with corresponding SOMs of these metabolites ranking in the top-*k* (*k* = 1, 3) position.

MetaPrint2D-React, which is one of the most commonly used methods for prediction of structures of metabolites, was selected to be compared with our method. Only test set 1 was employed to evaluate the prediction performances of RD-Metabolizer and MetaPrint2D-React, because test set 2 offered no information about the structures of metabolites. The prediction results for test set 1 are shown in Table 2. The two methods obtained similar performance in recall: 21.7% (RD-Metabolizer) and 20.6% (MetaPrint2D-React) of the metabolites were reproduced from the top-1 position. But RD-Metabolizer performed better than MetaPrint2D-React in precision: 30.6 and 22.9% of the predicted metabolites at rank 1 were experimentally observed. As a result, RD-Metabolizer exhibited superior performances than MetaPrint2D-React, which can be indicated by the value of *F*1-Measure. In addition, similar results could also be found from the top three ranking position. When interpreting the low values of recall and precision, the considerable variability in the metabolism reaction data for different parent molecules should be taken into account. Some compounds have been widely studied, resulting in the presence of more than 10 metabolites in test set 1. However, for the majority of compounds, only fewer than three metabolites have been reported.

**Table 2:** Prediction results of the metabolites for test set 1

Test set 1	Top-1				Top-3			
	Recall	Precision	*F*1-Measure	FP^a^	Recall	Precision	*F*1-Measure	FP^a^
RD-Metabolizer	0.217	0.306	0.254	802	0.355	0.241	0.287	1823
MetaPrint2D-React	0.206	0.229	0.217	1133	0.349	0.162	0.221	2953

^a^ FP is the total number of predicted false positive metabolites in the top-*k* (*k* = 1, 3) position for all molecules in test set 1

More importantly, the number of false positive metabolites generated by RD-Metabolizer was far lower than the number that generated by MetaPrint2D-React, indicating that we have already realized the anticipated purpose of developing RD-Metabolizer (Table 2). Some factors were responsible for the generation of false positive metabolites. RD-Metabolizer is one of the fingerprint-based data mining approaches, thus there may exist combination explosion problems for some reactions. For example, molecules containing phenolic hydroxyl group will be cleared from the body by making conjunction with one or more endogenous cofactors, such as glucose acid, sulfonic acid, amino acid, acetyl coenzyme A and glutathione. RD-Metabolizer was insensitive to the different chemical environments of the phenolic hydroxyl groups. It applied all conjugation reactions about phenolic hydroxyl groups in the databases for the query compound, and thus resulting in many unexpected metabolites. Therefore, it was extremely important for this category of metabolic reactions to be further refined and split by reaction SMARTS patterns to decrease the number of false positive metabolites. In additions, the incorrectly predicted SOMs also became the causes of the generation of unexpected metabolites. The accuracy of our method was largely influenced by the diversity of the fingerprint database we built. If the query molecule had some novel atomic fingerprint environments that are exactly the reaction centers, RD-Metabolizer would assign these atoms a normalized occurrence ratio of 0.0. Therefore, some other atoms in the molecule would have higher (than zero) normalized occurrence ratio and be top-ranked, even when the likelihood of their being a metabolic sites is very low [17]. Subsequently, some false positive metabolites would be generated.

### The influence of the number of fingerprint layers on prediction results

Compared with the 2D fingerprint similarity model built in SPORCalc (former version of MetaPrint2D) [43], an exact match operator was introduced to establish the fingerprint similarity model in our method. The exact match operator required that the corresponding top three rows in two fingerprint matrices are exactly the same. A site where metabolic reaction occurs was usually affected by its surrounding environment. Therefore, the introduction of the exact match operator was mainly to ensure the existence of small and identical surrounding environments for the reaction centers. Besides, the use of exact match operator for the top three layers of the fingerprint matrices was in accordance with the writing habit of the reaction SMARTS patterns for the fingerprint environments of the reaction centers, leading to improved computational efficiency.

**Fig. 5:**
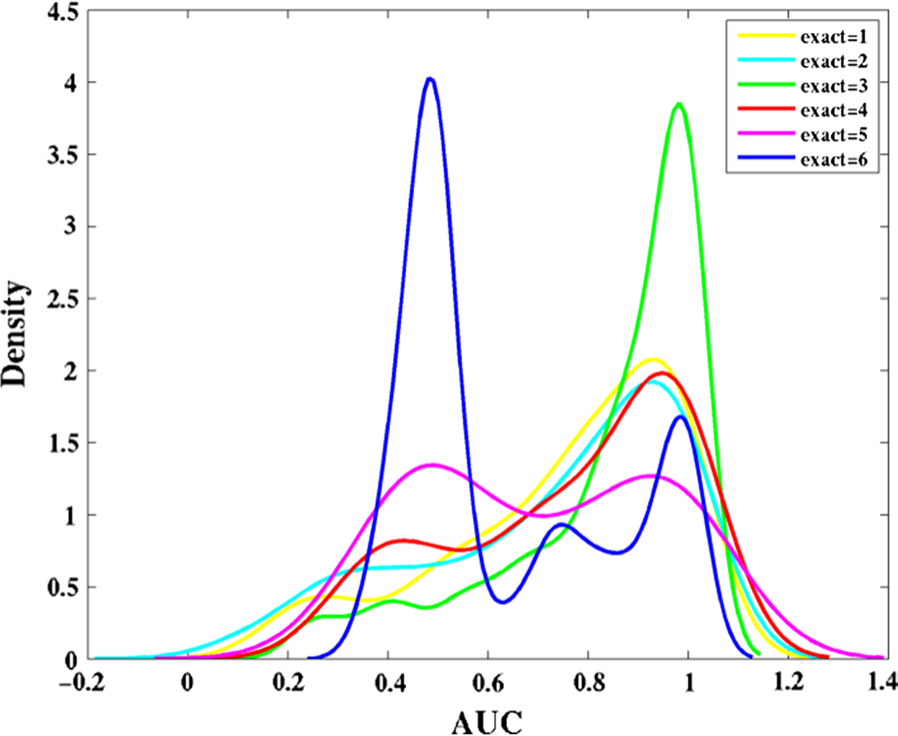
Kernel density estimation showing the changes in distribution of AUC scores for RD-Metabolizer predictions as the number of fingerprint levels to match exactly is varied

To explore whether it’s the optimal option to keep the top three rows of fingerprints the same for the exact match, we tested the performances of RD-Metabolizer with various layers of fingerprint to be identical using test set 2. The AUC value for each molecule was calculated, and the distributions of the AUC scores were analyzed by kernel density estimation [48, 49]. The kernel density estimation method analyzes the data distribution without using the prior knowledge of data distribution and without making any assumptions to data distribution. It studies data distribution from samples themselves, that’s why we selected this method to present the AUC distributions. We can clearly find that when the number of exact matching fingerprint levels is less (level = 1, 2, 3), the distribution is unimodal with the peak of AUC around 1.0, and exact match of the top three fingerprint levels has the highest probability density (Fig. 5). While the number of exact matching fingerprint levels is more (level = 4, 5, 6), the distribution is predominantly bimodal, with the peaks of AUC around 0.5 and 1.0. The estimation ability of RD-Metabolizer will be weakened, because such a search requiring exact matches to so many fingerprint levels returns little or no similar fingerprints for many of the atom environments in the test set, leading to an obvious peak of AUC around 0.5. The exact match of top one or two fingerprint levels provides little surrounding information, thus resulting in many false positive results generated by RD-Metabolizer. Therefore, their probability densities are lower than those of the exact match of top three fingerprints levels at the peak around 1.0. Overall, the results indicate that exact match of the top three fingerprint levels can bring best prediction results.

### Influence of molecular size

**Fig. 6:**
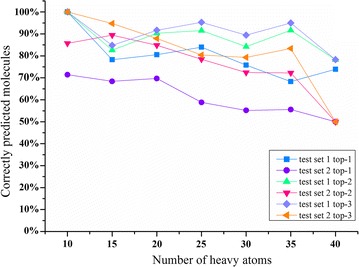
Percentage of correctly predicted molecules with respect to their size for test set 1 and test set 2

The prediction of SOMs becomes gradually difficult as the number of heavy atoms in a molecule increases. An ideal model would be able to correctly identify SOMs independent on the size of a molecule [37]. Therefore, we investigated the influence of molecular size on the prediction results of our method. Using the top-1 metric, the percentages of successfully predicted SOMs for the molecules from test set 1 and 2 both decreased as the molecular sizes increased (Fig. 6). When using the top-2 metric, the percentage of successfully predicted SOMs for the molecules from test set 1 went up slightly first, and then went down slightly, after the atom numbers are larger than 15. And the prediction accuracy reached its local peak when the atom numbers are increased to 35. A similar trend could also be observed using the top-3 metric, with more than 90 and 80% of the SOMs for the molecules from test set 1 and test set 2 being correctly predicted respectively. The results directly indicated that with the top-3 metric, RD-Metabolizer has a good predicting ability for drug-like molecules that have heavy atoms up to 35.

### Significance of detailed reaction SMARTS pattern

**Fig. 7:**
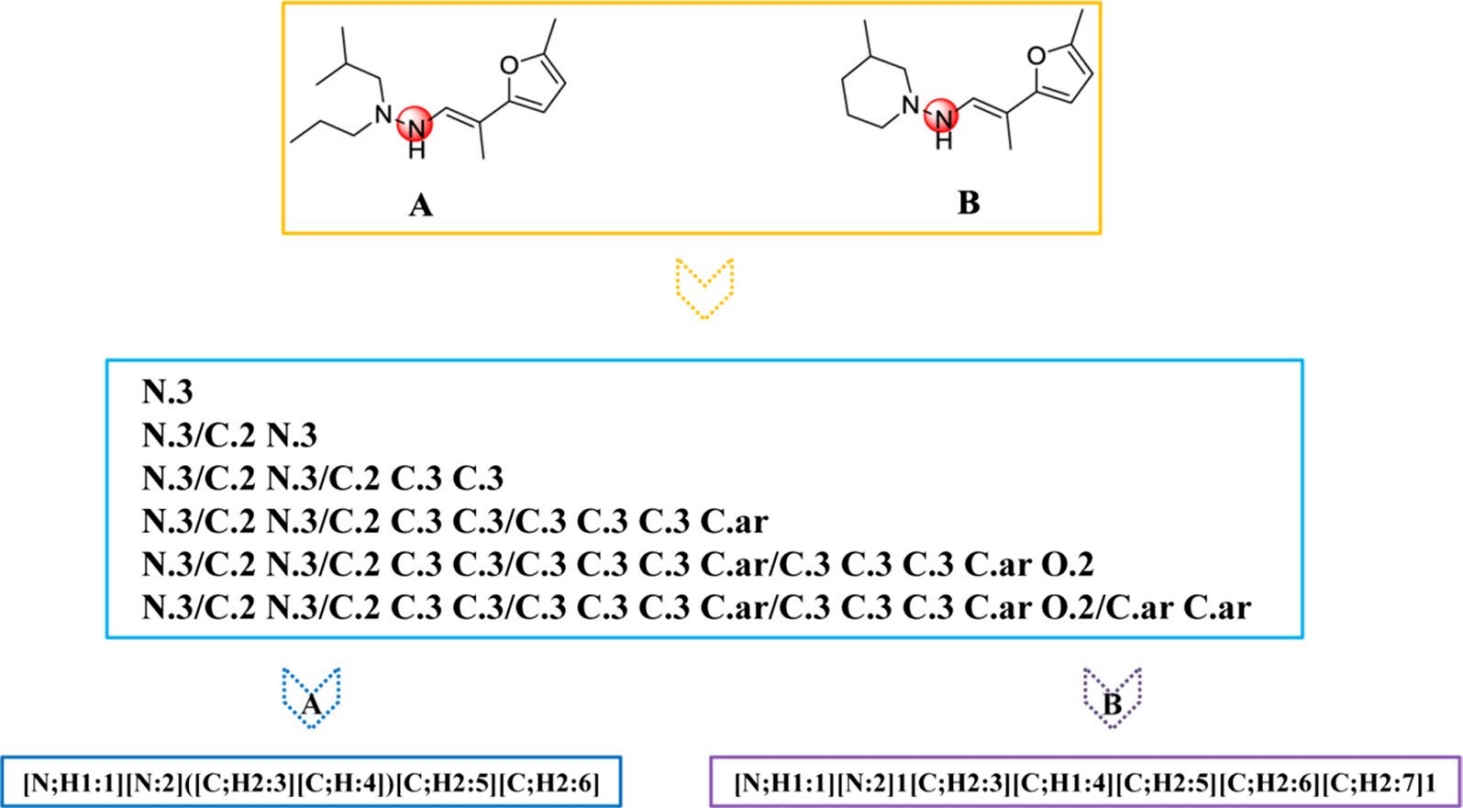
Illustration of the role of detailed reaction SMARTS pattern. The Molprint2D fingerprints of two N atoms that marked with *red circle* in compound A and B are the same, while the chemical environment of those two N atoms are dissimilar. The reaction SMARTS pattern is employed to make up the intrinsic defects of Molprint2D fingerprints, such as unable to identify the ring structures, therefore, those two N atoms are distinguished by different detailed reaction SMARTS patterns

**Fig. 8:**
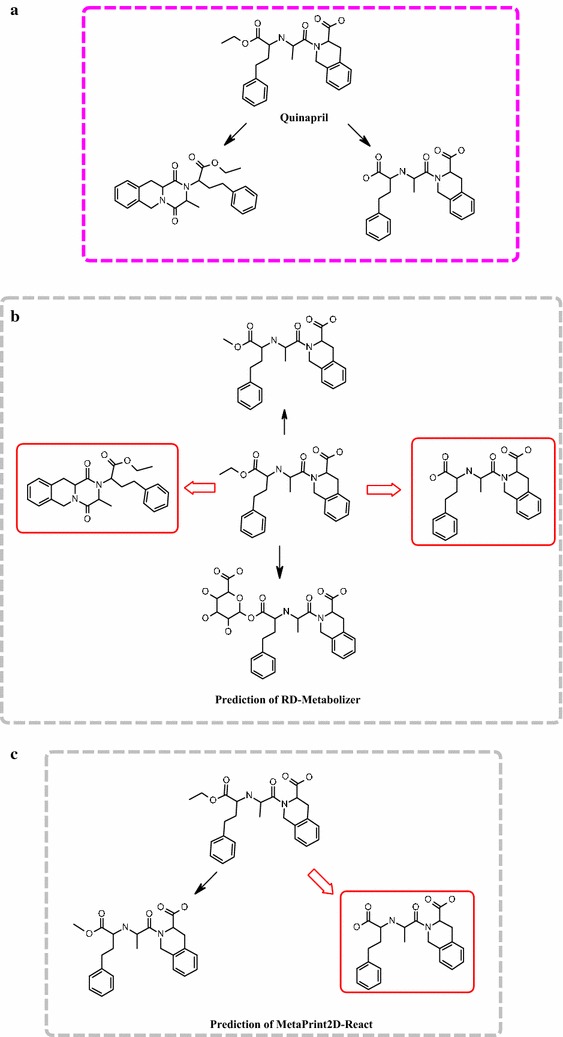
Comparison of prediction performance of RD-Metabolizer utilizing detailed reaction SMARTS pattern to generate structures of metabolites and MetaPrint2D-React using generic reaction SMARTS pattern to generate structures of metabolites. **a** The compound, Quinapril, has two metabolites determined by experiment: a hydrolysis product and a cyclization product. **b** The metabolites are generated by RD-Metabolizer and MetaPrint2D-React, respectively. The correctly predicted metabolites are marked with a *red border*. The prediction results of RD-Metabolizer based on the detailed reaction SMARTS pattern to generate structures of metabolites outperforms the prediction results of MetaPrint2D-React based on the generic reaction SMARTS pattern to generate structures of metabolites

In order to generate the structures of metabolites for the drug-like molecules, RD-Metabolizer needed to call two functions of RDKit to manipulate the pre-written detailed reaction SMARTS patterns. The detailed reaction SMARTS patterns contributed significantly to the prediction accuracy of RD-Metabolizer. To our knowledge, the current metabolites prediction methods usually utilized a generic reaction SMARTS pattern to represent a certain kind of metabolic reactions. For example, they used [*:1] ≫ [*:1]-[OH] to represent hydroxylation reaction. This is convenient to express simple metabolic reactions, but difficult to represent complex reactions in chemical reaction space, such as ring reaction types. Therefore, we employed the detailed reaction SMARTS pattern in our study. On one hand, it can make up the defects of Molprint2D fingerprint, which ignores H atom and is unable to identify ring structures. For example, the two N atoms labeled with red circles (Fig. 7) have dissimilar chemical environment, but the representations of Molprint2D fingerprint of those two N atoms are the same. If there is no clear differentiation, it will produce false positive results. However, the reaction SMARTS pattern enables flexible definitions for element, valence, aromaticity, charge, ring memberships of atoms, bond order and ring membership of bonds, and allows definition of metabolism reaction rules, which can describe specific chemical environment of reaction center. Therefore, the detailed reaction SMARTS patterns are employed to successfully distinguish those two N atoms labeled with red circle (Fig. 7). On the other hand, the detailed reaction SMARTS pattern can encode complex metabolism reaction types, such as ring reaction types (Table 3). It is reported that Quinapril has two metabolites (Fig. 8a), including Dioxopiperazine derivatives and quinaprilat [50, 51]. The two metabolites’ structures were successfully predicted by RD-Metabolizer (Fig. 8b), while MetaPrint2D-React only generated the quinaprilat one (Fig. 8c). This case demonstrated that by using the detailed reaction SMARTS pattern, RD-Metabolizer is capable to deal with more complex metabolic reaction types and will no doubt has a broader application.

**Table 3:** Examples of the expressions of detailed reaction SMARTS pattern for complex ring reactions

Metabolism reactions	Reaction SMARTS pattern
(Cyclization)	[C:1]([NH2])[C:2][C:3][C:4](=O)O >>[C:1]1[C:2][C:3][C:4](=O)N1
(Ring opening)	[C:1][N:2]1[C:3][C:4][C:5][C:6][C:7]1 >>[C:1][N:2][C:3][C:4][C:5][C:6][C:7](=O)O
(Ring contraction)	[c:1]1[c:2][C:3]=[N:4][C:5](O)[C:6](=O)[N:7]1 >>[c:1]1[c:2][C:3]=[N:4][C:5](=O)[N:7]1.[C:6]
(Ring expansion)	[C:1]1[C:2][C:3][C:4][C:5]1(O)(C#C) >>[C:1]1[C:2][C:3][C:4]C[C:5]1(O)

### Case study

**Fig. 9:**
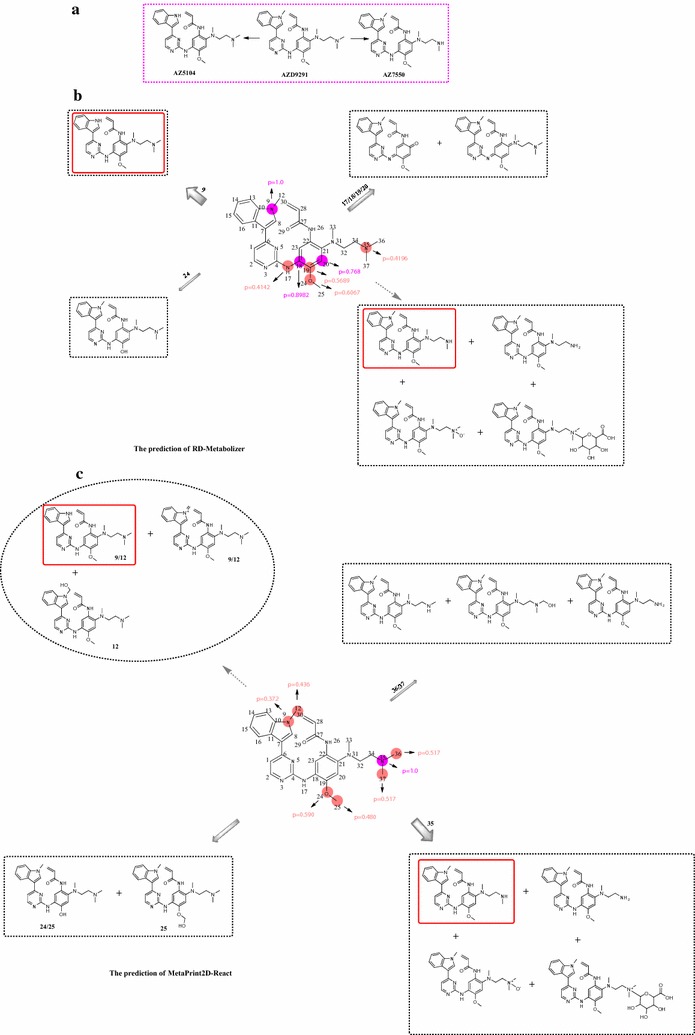
Prediction of SOMs and metabolites for AZD9291 and comparison of the integrated prediction performance of RD-Metabolizer and MetaPrint2D-React. **a** The experimental metabolism data of AZD9291. **b** The predicted results are generated by RD-Metabolizer. **c** The predicted results are generated by MetaPrint2D-React. The sites with metabolic probability ranging from 0.33 to 1.00 are labeled by color-coded circles and the corresponding values of metabolic probability are also labeled on the structure. The correctly predicted metabolites are marked with a *red border* and the width of the *arrows* indicates the metabolic probability scale of sites in the molecule

RD-Metabolizer was applied to the compound AZD9291 to further illustrate its practical application in medicinal chemistry. AZD9291 (Osimertinib) is a novel, selective third-generation irreversible inhibitor of Epidermal Growth Factor Receptor (EGFR), which can overcome T790M-mediated resistance. A survey of the literatures available showed that AZD9291 was metabolized into two metabolite species: AZ5104 and AZ7550 (Fig. 9a) [32, 52]. Among them, AZ5104 is the main metabolite and acts as another potent inhibitor of EGFR. The results of prediction illustrate that the two metabolites AZ5104 and AZ7550 can be found by RD-Metabolizer with the corresponding metabolic probabilities of 1.00 and 0.42 (Fig. 9b). Besides, different predicted SOMs are distinguished by different colored circles according to the metabolic probability division rules of RD-Metabolizer. By calculation, the top-3 prediction precision and recall of RD-Metabolizer are respectively 33.3 and 50%, while the top-3 prediction precision and recall calculated by MetaPrint2D-React are respectively 16.7 and 50%. Thus it is proved directly that the number of false positive metabolites generated by RD-Metabolizer is lower than that generated by MetaPrint2D-React. In addition, AZ5104 can be precisely ranked in the top-1 prediction position of RD-Metabolizer, while the top-1 prediction position of MetaPrint2D-React is AZ7550. Collectively, the prediction results of RD-Metabolizer adjusted by the detailed reaction SMARTS patterns are superior to the prediction results of MetaPrint2D-React. In MetaPrint2D-React, one or two neighboring atoms of potential SOMs are also treated as reaction center atoms (Fig. 9c). For example, for the N-dealkylation reaction, MetaPrint2D-React generally flags the nitrogen and the connected carbon atoms as potential SOMs. MetaPrint2D-react thinks that flagging one or two neighboring atoms of potential SOMs can provide valuable hints about which metabolic reactions may take place. However, from the prediction results of MetaPrint2D-React, the metabolic probability of the carbon atom (C12) in the indole *N*-methyl group is higher than the nitrogen atom (N9), and the corresponding metabolites of C12 contain not only the metabolites of N9 but also a hydroxylated metabolite. This inevitably leads to data redundancy and affects the final ranking of the predicted SOMs. Besides, it is difficult for MetaPrint2D-React to distinguish between the main metabolite and the subordinate metabolite, because N35 rather than N9 has ranked first in the SOMs list predicted by MetaPrint2D-React. Nevertheless, these situations do not exist in RD-Metabolizer, suggesting itself as an accurate and highly efficient toolkit for chemist and medicinal chemists.

## Conclusion

This work described RD-Metabolizer, an integrated, low false positive and reaction types extensive approach to predict metabolic sites and metabolites of drug-like molecules. The detailed reaction SMARTS patterns were firstly employed to encode different metabolism reaction types with the aim of covering larger chemical reaction space. RDKit was utilized to act on pre-written reaction SMARTS patterns to correct the metabolic ranking of each site in a molecule generated by the 2D fingerprint similarity calculation model as well as to generate the corresponding structures of metabolites. These are critical procedures, as they can meet the integrated and low false positive goals. By comparing with other widely used methods, it is found that RD-Metabolizer has better or comparable performance in predicting SOMs and produces fewer false positive metabolites. In addition, a specific example concerning AZD9291, which is a mutant-selective EGFR inhibitor, was conducted to further illustrate the prediction accuracy and efficiency of RD-Metabolizer. In summary, RD-Metabolizer will serve as a useful toolkit for the early metabolic properties assessment of lead compounds and drug candidates at the preclinical stage of drug discovery.

## Abbreviations

SOMs: site of metabolism
MIFs: molecular interaction fields
DFT: density functional theory
RS-predictor: RegioSelectivity-predictor
SVM: support vector machine
MS: mass spectrometry
LC–MS/MS: liquid chromatography/tandem mass spectrometry
EGFR: Epidermal Growth Factor Receptor
